# A better way to determine sample size to detect changes in length of mechanical ventilation?

**DOI:** 10.1186/cc14307

**Published:** 2015-03-16

**Authors:** YS Chiew, C Pretty, D Redmond, GM Shaw, T Desaive, JG Chase

**Affiliations:** 1 University of Canterbury, Christchurch, New Zealand; 2 Christchurch Hospital, Christchurch, New Zealand; 3 University of Liege, Belgium

## Introduction

Estimation of effective sample size (*N*/arm) is important to ensure power to detect significant treatment effects. However, traditional parametric sample size estimations depend upon restrictive assumptions that often do not hold in real data. This study estimates *N *to detect changes in length of mechanical ventilation (LoMV) using Monte-Carlo simulation (MCS) and mechanical ventilation (MV) data to better simulate the cohort.

## Methods

Data from 2,534 MV patients admitted to Christchurch Hospital ICU from 2011 to 2013 were used. *N *was estimated using MCS to determine a sample size with power of 80%, and compared with the Altman's nomogram for two patients groups, (1) all patients and (2) targeted patients with 1 <LoMV ≤15 days. MCS allows any range of intervention effect to be simulated, where this study tested a 10 and 25% difference in LoMV (0.5 to 1.25 days for mean LoMV of 5 days). The simulated LoMV for the intervention group is compared with the LoMV in a control group using the one-sided Wilcoxon rank-sum test, Student *t *test, and Kolmogorov-Smirnov test to assess central tendency and variation.

## Results

**Figure 1:**
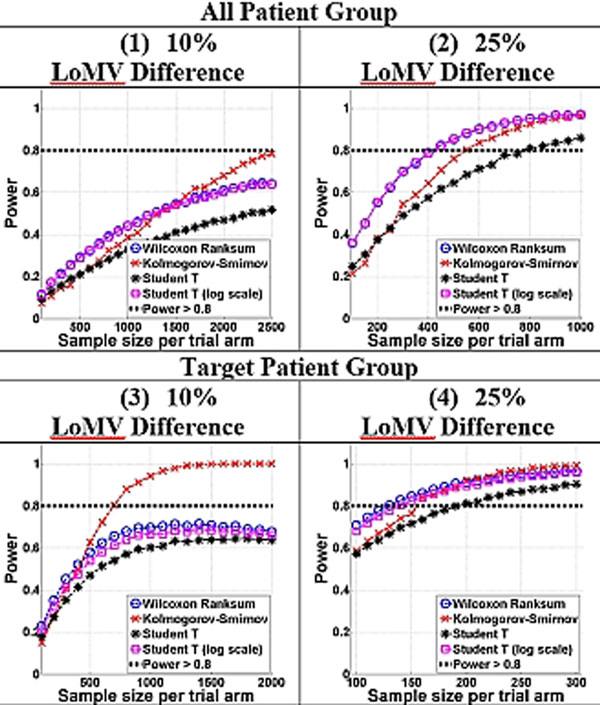


The distribution of LoMV is heavily skewed. Altman's nomogram assumes a normal distribution and found *N *>1,000 to detect a 25% LoMV change. Figure 1 panels (1) and (2) show *N *for 80% power if all patients were included, and panels (3) and (4) for the targeted patient group. Panels (1) and (3) show that it is impossible to achieve 80% power for a 10% intervention effect. For 25% effect, MSC found *N *= 400/arm (all patients) and *N *= 150/arm (targeted cohort).

## Conclusion

Traditional parametric sample size estimation may overestimate the required patients. MCS can estimate effective *N*/arm and evaluate specific patient groups objectively, capturing local clinical practice and its impact on LoMV. It is important to consider targeting specific patient groups by applying patient selection criteria that can be easily translated into trial design.

